# Intranasal oxytocin modulates brain responses to voice-identity recognition in typically developing individuals, but not in ASD

**DOI:** 10.1038/s41398-020-00903-5

**Published:** 2020-07-07

**Authors:** Kamila Borowiak, Katharina von Kriegstein

**Affiliations:** 1 Technische Universität Dresden, Bamberger Straße 7, 01187 Dresden, Germany; 2 Max Planck Institute for Human Cognitive and Brain Sciences, Stephanstraße 1a, 04103 Leipzig, Germany; 3 Berlin School of Mind and Brain, Humboldt University of Berlin, Luisenstraße 56, 10117 Berlin, Germany

**Keywords:** Neuroscience, Psychology, Autism spectrum disorders

## Abstract

Faces and voices are prominent cues for person-identity recognition. Face recognition behavior and associated brain responses can be enhanced by intranasal administration of oxytocin. It is unknown whether oxytocin can also augment voice-identity recognition mechanisms. To find it out is particularly relevant for individuals who have difficulties recognizing voice identity such as individuals diagnosed with autism spectrum disorder (ASD). We conducted a combined behavioral and functional magnetic resonance imaging (fMRI) study to investigate voice-identity recognition following intranasal administration of oxytocin or placebo in a group of adults diagnosed with ASD (full-scale intelligence quotient > 85) and pairwise-matched typically developing (TD) controls. A single dose of 24 IU oxytocin was administered in a randomized, double-blind, placebo-controlled and cross-over design. In the control group, but not in the ASD group, administration of oxytocin compared to placebo increased responses to recognition of voice identity in contrast to speech in the right posterior superior temporal sulcus/gyrus (pSTS/G) — a region implicated in the perceptual analysis of voice-identity information. In the ASD group, the right pSTS/G responses were positively correlated with voice-identity recognition accuracy in the oxytocin condition, but not in the placebo condition. Oxytocin did not improve voice-identity recognition performance at the group level. The ASD compared to the control group had lower right pSTS/G responses to voice-identity recognition. Since ASD is known to have atypical pSTS/G, the results indicate that the potential of intranasal oxytocin to enhance mechanisms for voice-identity recognition might be variable and dependent on the functional integrity of this brain region.

## Introduction

Recognition of others has an essential role for successful social interactions. Correct recognition of identity determines communication behavior such as content and style of a conversation and allows for appropriate actions in social contexts. Impairments of person-identity recognition can contribute to difficulties in communication and avoidance of social situations^1,2^.

A large research community has investigated the potential of intranasal oxytocin administration to augment different aspects of social behavior including perception of faces (for review see ref. ^3^). Oxytocin is a nine-amino acid neuropeptide that is critical for the regulation of social behaviors^4,5^. Intranasal oxytocin enhanced the ability to recognize the emotional state and the identity of others from the face in TD individuals^6–9^ and individuals with a selective face-identity recognition deficit^10^. It remains unknown whether intranasal oxytocin can augment recognition of person identity from the voice.

Several clinical conditions are affected by impairments in person-identity recognition from voices including ASD, schizophrenia and selective person-identity recognition deficits^11–13^ (for review see ref. ^14^). ASD is a clinical condition characterized by core difficulties in social interaction and communication (DSM-5^15^). Compared to TD individuals, adults with ASD without intellectual impairment have difficulties with differentiating between voices, and to learn and recognize unfamiliar voices, while recognition of famous voices remains relatively typical^16–18^, but see^19^. The voice-identity recognition difficulties in ASD are associated with impaired perception of acoustic voice features such as vocal pitch^16,20^. In contrast, speech recognition is relatively intact in ASD without intellectual impairment, at least in situations with a good signal-to-noise ratio^18,21–23^. Current evidence suggests that intranasal oxytocin can enhance some aspects of face and voice perception in ASD including orienting responses toward faces and voices^24–26^ or emotion recognition from them^27–29^. Currently, it is an open question whether intranasal administration of oxytocin can boost recognition of voice identity in ASD.

One possible mechanism by which oxytocin influences behavior during face and voice perception might be an increase of brain responses in regions sensitive to faces and voices. Intranasal oxytocin modulated responses to emotional faces in visual sensory regions implicated in face perception in TD individuals^29–31^ and in individuals with ASD^29,32^, but see ref. ^33^. Responses to emotional voices in auditory sensory regions - including the superior temporal sulcus/ gyrus (STS/G) - were higher after oxytocin compared to placebo administration in ASD^34^.

In ASD, voice-identity recognition difficulties are thought to be—at least partly—based on atypical mechanisms in the posterior (p)STS/G^18^. Parts of the STS/G house the so-called temporal voice-sensitive areas (TVA). The TVAs show higher responses to vocal compared to non-vocal sounds^35,36^ and are recruited for recognition of voice identity compared to speech in TD individuals^37,38^. The right pSTS/G has been associated with perceptual analysis of voice-identity information, where acoustic voice features are integrated into a coherent percept^39,40^. A previous study showed that the right pSTS/G responses to voice-identity recognition are reduced in ASD compared to TD^18^. This fits well with the behavioral profile of the voice-identity recognition in ASD pointing toward a perceptual nature of the difficulties^16^. That communication difficulties and person-identity recognition difficulties in ASD might be partly based on atypical perceptual mechanisms is a relatively novel view on the disorder^41–43^. Recent studies indicated that difficulties in recognizing dynamic faces are also likely due to atypical processing at the level of perception^44,45^. In other views, impaired person-identity recognition in ASD has been linked to impaired mnemonic mechanisms^46,47^ or to decreased social interest^48^.

Here, we systematically investigated whether voice-identity recognition can be enhanced by intranasal oxytocin administration in adults with ASD without intellectual impairment and pairwise-matched TD controls. All participants received a single dose of oxytocin or placebo in two sessions with fMRI. Participants learned novel voices and subsequently performed a speaker recognition task and a speech recognition task on the same stimuli in the MRI environment^37^. The experiment tested the ability to recognize recently learned voices across varying speech information, which represents one aspect of person-identity recognition from the voice^49^. Given that speech recognition ability is relatively typical in adults with ASD without intellectual impairment in contrast to impaired voice-identity recognition (e.g^17,18^), the speech task was a suitable control task to evaluate if participants understood the experiment and paid attention to it. In addition, contrasting the speaker task to the speech task allowed for specifically targeting mechanisms underlying voice-identity processing in contrast to processing other vocal information. Previous research had shown that the voice-identity recognition experiment reveals TVA responses to recognition of voice identity in TD (e.g. refs. ^37,38^). Furthermore, it had been used to assess differences in voice-identity processing between TD individuals and individuals with ASD both on the behavioral as well as neural level^18^.

Given previous reports on enhanced face-identity recognition and increased brain responses to faces due to oxytocin in TD individuals^8,29^, we predicted that oxytocin compared to placebo administration might increase speaker recognition accuracy and/or right pSTS/G responses to speaker in contrast to speech recognition, at least in TD controls. In ASD, it was unclear whether oxytocin can elevate speaker recognition accuracy and modulate the right pSTS/G responses, as no previous study had investigated oxytocin-related modulation of face-identity recognition in ASD and the right pSTS/G showed atypical functioning during voice-identity processing in ASD compared to TD^18^.

## Methods

### Participants

The study sample included 18 TD individuals (control group) and 18 individuals diagnosed with ASD (ASD group), who were pairwise-matched on gender, chronological age, handedness^50^ and full-scale intelligence quotient (IQ) (Table 1; Supplementary Methods—Participants).

**Table 1:** Descriptive statistics for the control group, the ASD group and group comparisons.

	Control		ASD		
Gender	15 males, 3 females		15 males, 3 females		
Handedness^a^	16 right, 2 left		16 right, 2 left		
	*M*	SD (range)	*M*	SD (range)	*p*
Age	29.44	6.92 (22-45)	30.17	7.64 (22-47)	0.768
WAIS^b^ subscales					
Full-scale IQ	115.22	9.98 (95–130)	112.89	13.23 (97–136)	0.554
Verbal comprehension	115.39	9.71 (95–134)	112.78	11.03 (93–137)	0.456
Perceptual reasoning	106.61	7.52 (94–123)	110.94	13.76 (81–131)	0.249
Working memory	107.50	15.29 (86–138)	116.00	15.69 (91–146)	0.109
Processing speed	112.72	19.14 (86–147)	101.53	14.67 (83–135)	0.062
Concentration (d2)^c^	106.50	13.42 (84–130)	100.11	10.49 (84–130)	0.121
AQ^d^	16.89	3.27 (11–21)	39.28	7.81 (14–47)	0.001^*^

*M* mean, *SD* standard deviation.

*Significant group differences (*p* < 0.05).

^a^Handedness was assessed using the Edinburgh Handedness Inventory^50^.

^b^WAIS = German adapted version of the Wechsler Adult Intelligence Scale;^52,54^*M* = 100; *SD* = 15.

^c^Test d2 (KL); *M* = 100; *SD* = 10^55^.

^d^Q = Autism Spectrum Quotient^61^.

All participants had an IQ within the normal range or above (defined as a full-scale IQ of at least 85). Pairs of control and ASD participants were considered matched on IQ if the full-scale IQ difference within each pair was maximally one standard deviation (15 IQ points). IQ was assessed using the Wechsler Adult Intelligence Scale (WAIS-III^51^; German adapted version^52^; WAIS-IV^53^; German adapted version^54^; Supplementary Methods—Participants). The control and the ASD group had comparable concentration performances (d2 test of attention^55^; Table 1). All participants reported to have normal or corrected to normal vision and had normal hearing levels. We formally assessed hearing levels via pure-tone audiometry (250–8000 Hz) using a screening audiometry (Micromate 304; Madsen, Denmark). All participants were native German speakers and were free of psychostimulant medication.

All participants with ASD had previously received a formal clinical diagnosis of Asperger Syndrome according to the diagnostic criteria of the International Classification of Diseases (ICD-10^56^). The diagnosis was additionally confirmed based on the Autism Diagnostic Observation Schedule^57^ (German version^58^), that was conducted in the context of clinical diagnostics or by trained researchers. If caregivers or relatives were available (*n* = 9), we also performed the Autism Diagnostic Interview-Revised^59^ (German version^60^). Four ASD participants had previously received a formal clinical diagnosis of comorbid psychiatric disorders (social anxiety, depression (remitted), and posttraumatic stress disorder) according to the diagnostic criteria of the ICD-10^56^. Control participants were screened for presence of autistic traits. None of them met a clinically relevant extent according to the Autism Spectrum Quotient (cut-off score = 32) (AQ^61^, Table 1). None of the control participants reported a history of psychiatric disorders or a family history of ASD. None of the participants reported any history of neurological or endocrine disease. All participants received monetary compensation after study completion.

### Experimental procedure

The experimental procedure was approved by the Ethics Committee of the Medical Faculty at the University Leipzig (403-13-16122013). All participants gave written informed consent and were familiarized with the nasal spray administration and the experimental procedure.

**Fig. 1:**
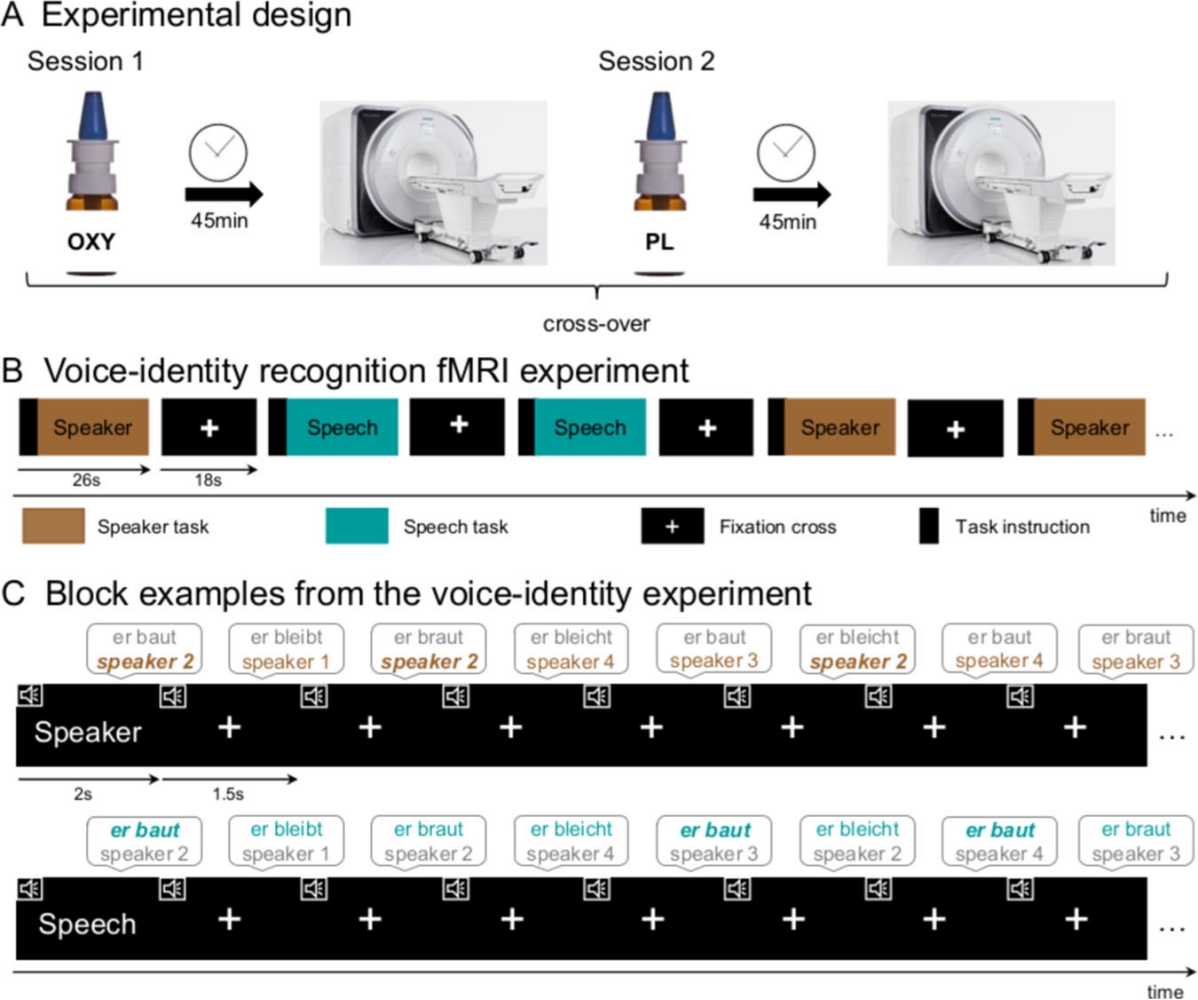
Study overview. **a** Experimental design of the study. All participants self-administered oxytocin and placebo in form of a nasal spray at the beginning of two fMRI sessions. The two fMRI sessions were separated by a 4-week interval. Forty-five minutes after receiving the substance, participants performed the voice-identity recognition fMRI experiment in the MRI environment. Before and after the substance administration participants filled out the Multidimensional Mood State Questionnaire (MDBF^51^) assessing their current mood, wakefulness and calmness. Twenty-four hours prior to MRI scanning, participants were instructed to abstain from alcohol, nicotine, and caffeine. **b** During the voice-identity recognition experiment, participants listened to blocks of auditory two-word sentences spoken by four speakers. In one task, participants had to recognize who was speaking (speaker task). In the other task, participants had to recognize what was said (speech task). **c** At the beginning of each block, a written word instructed participants to perform one of the tasks (“speaker” for the speaker task or “speech” for the speech task). For each sentence within the block participants had to decide if it was spoken by the target speaker (here “speaker 2” in the speaker task) or whether it matched the content of the target sentence (here “er baut” in the speech task). The stimuli for the two tasks were identical. OXY oxytocin, PL placebo.

Two fMRI sessions were conducted in a randomized, double-blind, placebo-controlled, within-subject, cross-over design separated by 4 weeks (Fig. 1a; Supplementary Methods—Experimental design). In each session, all participants self-administered oxytocin (24 IU, Syntocinon-Spray, Novartis, Basel, Switzerland) or placebo via nasal spray under supervision of the study coordinator and in accordance with the latest standardization guidelines^62^. Voice-identity recognition fMRI experiment was started 45 min after substance administration and took ~42 min. Oxytocin compared to placebo administration had no substantial influence on participants’ reported mood, wakefulness, and calmness as assessed using Multidimensional Mood State Questionnaire (MDBF^63^; Supplementary Methods—Experimental design; Supplementary Table S1).

### Voice-identity recognition experiment

Participants were first familiarized with the speakers’ voices and the experimental tasks on a laptop outside the MRI environment (Supplementary Methods—Voice-identity recognition experiment). Subsequently, recognition of speakers’ voice identity was tested in the MRI scanner (voice-identity recognition fMRI experiment).

### Voice-identity recognition fMRI experiment

The experiment was a 2 × 2 × 2 factorial design with the within-subject factors *Task* (speaker, speech) and *Substance* (placebo, oxytocin), and the between-subject factor *Group* (control, ASD). The stimulus material consisted of 128 auditory-only two-word sentences spoken by eight professional male German native speakers. Stimuli were divided into two stimulus sets to avoid learning effects between the fMRI sessions. Each stimulus set included 64 different sentences from four speakers and both stimulus sets had a comparable difficulty level (Supplementary Methods—Voice-identity recognition experiment).

The experimental trials were grouped into blocks. In each block, participants performed either the speaker task or the speech task (Fig. 1b). At the beginning of each block (Fig. 1c), participants received a task instruction. They saw a written instruction screen “speaker” or “speech” to announce which task to perform, and simultaneously heard a sentence spoken by one of the four speakers (i.e., target). This was followed by a stream of 16 sentences spoken by one of the four speakers (i.e., test trials). The test trials were four sentences that were phonologically similar (e.g., “Er baut” (English: “He builds”), “Er braut” (English: “He brews”), “Er bleibt” (English: “He stays”), “Er bleicht” (English: “He bleaches”)). The test trials were presented in a random order. For the speaker task, participants memorized the target speaker and indicated for each test trial whether the speaker matched the target speaker or not, independent of the content of the sentence. For the speech task, participants memorized the content of the target sentence and decided for each test trial whether the content matched the target sentence or not, independent of the speaker. Each block was followed by a silence block (18 s) during which a fixation cross was presented on the screen. Each block was presented twice: once in the speaker task and once in the speech task, so that the stimuli were the same for both task conditions. Each task condition included 28 blocks (56 blocks in total) and 448 trials (896 trials in total) presented in a pseudorandom order. In each trial (1.5 s), one test sentence was presented for ~1 s and the response could be given until the end of the trial (1.5 s long response window). Responses were made via a button box. The experiment was divided into four fMRI runs of ~10 min each.

### MR Image acquisition

Functional and structural MR data were acquired on a SIEMENS MAGNETOM Prisma (3 Tesla MRI scanner (Siemens, Germany)). Functional images were collected with a 20-channel head coil using a multi-band accelerated echo planar imaging sequence with a multi-band factor 3 (TR = 2000 ms, TE = 23 ms, flip angle = 90°, 60 slices, whole brain coverage, slice thickness = 2.5 mm, interslice gap = 0.25 mm, in-plane resolution = 2 × 2 mm). Images were acquired with a slice tilt of *β* = −15° to improve Blood Oxygenation Level Dependent (BOLD) sensitivity in temporal regions^64^ (Supplementary Methods—MRI data acquisition and analysis).

### Data analysis

Behavioral data were analyzed with PASW Statistics 24.0 (IBM SPSS Statistics, USA). The behavioral data were tested for normality and homogeneity of variance using the Shapiro–Wilk test and the Levene’s test, respectively. The behavioral data followed normal distribution, except for the performance of the ASD group in both tasks in the placebo condition (both *p*s ≤ 0.010) and in the speech task in the oxytocin condition (*p* = 028), and in the control group in the speech task in the oxytocin condition (*p* = 0.005). Data variance was equal between the groups (all *p*s > 0.123). We assessed differences between the substance conditions and the groups using analyses of variance (ANOVA). Level of significance was defined at *α* = 0.05. MRI data were analyzed using Statistical Parametric Mapping (SPM 12; Wellcome Trust Centre of Imaging Neuroscience, London, UK; http://www.fil.ion.ucl.ac.uk/spm) in a Matlab environment (version 10.11, The MathWorks, Inc., MA, USA). For details, see Supplementary Methods—MRI data acquisition and analysis and Supplementary Table S2.

### Hypotheses

Behavioral and fMRI data analysis was based on a statistical model with factors *Task* (speaker, speech), *Substance* (placebo, oxytocin) and *Group* (control, ASD). First, we tested the main hypotheses about the modulation of voice-identity recognition by intranasal oxytocin. In controls, we hypothesized an increase of speaker recognition accuracy and right pSTS/G responses to “speaker > speech” (i.e., a significant interaction *Task* x *Substance* in controls). In ASD, we had two alternative hypotheses. First, if the reduced right pSTS/G responses to voice-identity recognition are due to atypicalities in this region, oxytocin cannot elevate speaker recognition accuracy and the right pSTS/G responses in ASD (i.e., there should be a significant interaction *Task* × *Substance* × *Group* and a significant interaction *Task* × *Substance* in controls only). If the reduced right pSTS/G responses are due to atypical processing in another region, then oxytocin can elevate speaker recognition accuracy and right pSTS/G responses in ASD (i.e., there should be a significant interaction *Task* × *Substance* in ASD similar to controls). Second, we tested for replication of previous findings that right pSTS/G responses to the contrast “speaker > speech” are lower in ASD than in controls (i.e., *Task* × *Group*). Finally, we explored if the right pSTS/G responses to “speaker > speech” were positively correlated with speaker recognition accuracy.

### Significance thresholds for fMRI second-level analyses

Following the hypothesis-driven approach, effects were considered significant at *p* < 0.05 family wise error (FWE) corrected for a region of interest (ROI). This approach has been suggested to be suitable for studies with relatively small sample sizes, because it reduces the number of multiple comparisons (i.e., the number of voxels)^65,66^. We created two ROIs in the right pSTS/G. The first pSTS/G-ROI encompassed portions of the right pSTS/G that had responded to “speaker > speech” in TD individuals in previous studies^18,38,67^ (Supplementary Fig. S1A). The second pSTS/G-ROI included a portion of the right pSTS/G that had showed significantly reduced responses to “speaker > speech” in ASD compared to TD individuals^18^ (Supplementary Figure S1B). We used the first pSTS/G-ROI for the small volume correction (SVC) of the contrast “speaker > speech” within each group separately. The second pSTS/G-ROI was used for SVC of response differences to “speaker > speech” between the substance conditions (*Task* × *Substance*) and between the groups (*Task* × *Group*). Effects outside the ROIs were considered significant at *p* < 0.05 FWE corrected for the whole brain.

### Correlation analysis

We explored if the right pSTS/G responses to “speaker > speech” were behaviorally relevant for voice-identity recognition. We extracted parameter estimates of the contrast “speaker > speech” from the second pSTS/G-ROI (Fig. S1B) and correlated them with speaker recognition accuracy using the Pearson correlation. This was done in PASW Statistics 24.0 for each group and substance condition separately. We chose the second pSTS/G-ROI to assess if potential response differences between the substance conditions and the groups were related to behavioral performance. We report Pearson’s *r* as an estimate of the effect size of the correlations. Moreover, we assessed if the brain-behavior correlation coefficients significantly differed between the substance conditions and the groups using Fisher’s *r* to z transformation.

## Results

### Voice-identity recognition accuracy was not modulated by oxytocin in any group

We conducted a repeated measures ANOVA on the percent-correct scores with the within-subject factors *Task* (speaker, speech) and *Substance* (placebo, oxytocin), and the between-subject factor *Group* (control, ASD). There was a main effect of *Task* (*F*(1,34) = 15.349, *p* < 0.001, *η*^2^ = 0.311), so that the speaker task was significantly more difficult than the speech task in both groups (Supplementary Fig. S2A; Supplementary Table S3). We found a significant main effect of *Group* (*F*(1,34) = 4.229, *p* = 0.047, *η*^2^ = 0.111) indicating that the ASD group compared to the control group had significantly lower performance across both tasks and substance conditions. No other effects reached significance (all *p*s > 0.05). There were no significant substance or group effects for response times (Supplementary Results—Response times, Supplementary Fig. S2B; Supplementary Table S3).

### Right pSTS/G responses to speaker task compared to speech task in the control and ASD group

**Fig. 2:**
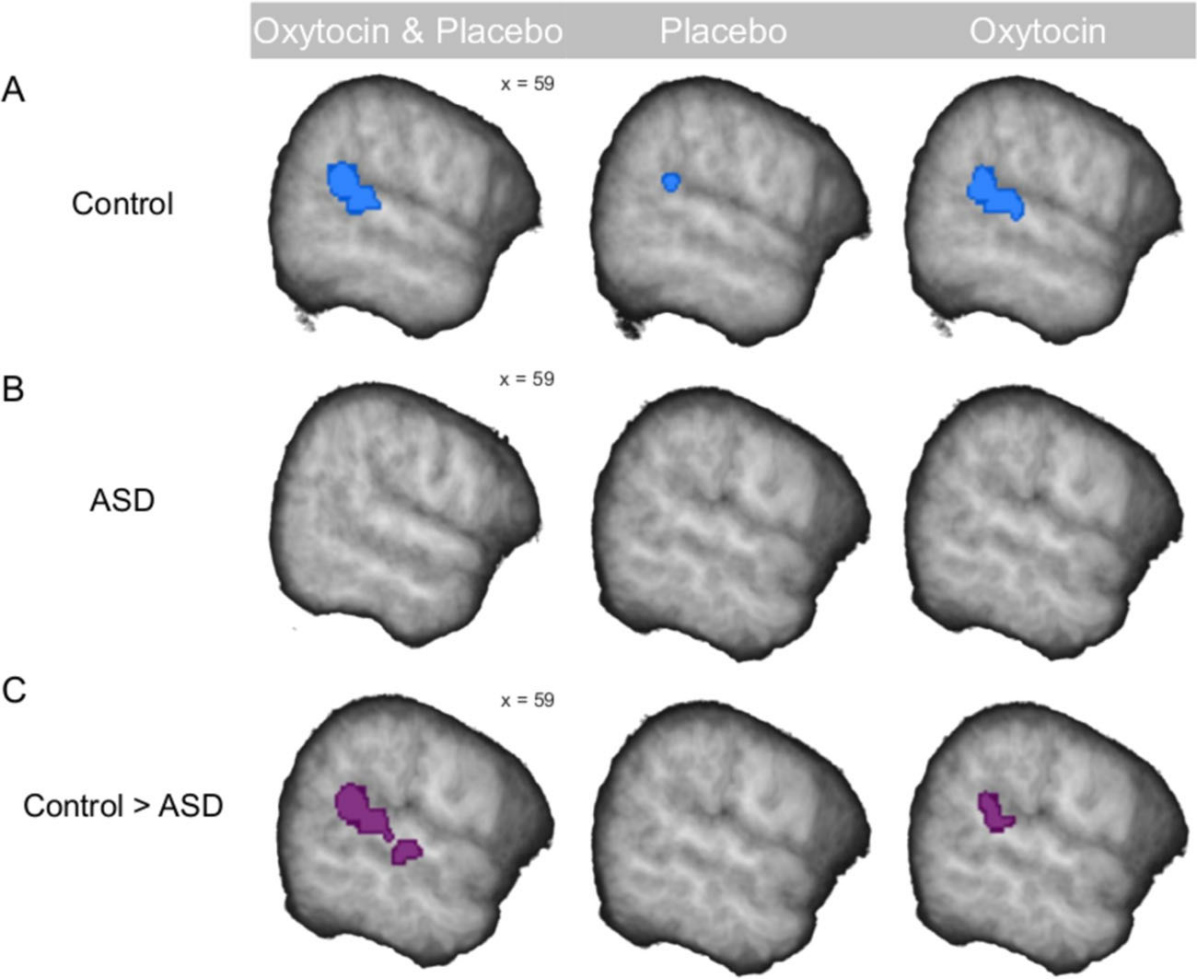
Brain responses in the right pSTS/G to the contrast “speaker > speech”. **a** The control group showed significantly higher BOLD response when recognizing speaker compared to speech across both substance conditions, in the oxytocin condition and in the placebo condition. **b** The ASD group did not have any significant BOLD response when recognizing speaker in comparison to speech in any of the substance conditions. **c** The control group compared to the ASD group had significantly higher BOLD response to recognition of speaker compared to speech across both substance conditions and in the oxytocin condition. There were no such differences in the placebo condition. The effects were significant at *p* < 0.05 FWE corrected for the ROI. For display purposes only, within-group effects are presented at the threshold of *p* = 0.01 uncorrected within the ROI, and between-group effects at the threshold of *p* = 0.05 uncorrected within the ROI. All results were overlaid onto a sample specific average image of normalized T1-weighted structural images. x = MNI coordinate.

We first analyzed responses in the right pSTS/G to the contrast “speaker > speech” in each group separately. The control group showed significant right pSTS/G responses across both substance conditions and in both substance conditions separately (Fig. 2a) (*p* < 0.05 FWE corrected for ROI; Supplementary Table S4). In the ASD group, there were no such significant responses (Fig. 2b). Only at a lenient statistical threshold, right pSTS/G responses appeared when pooled over both substance conditions (*p* = 0.007 uncorrected).

### Oxytocin increased right STS/G responses in controls, but not in ASD

**Fig. 3:**
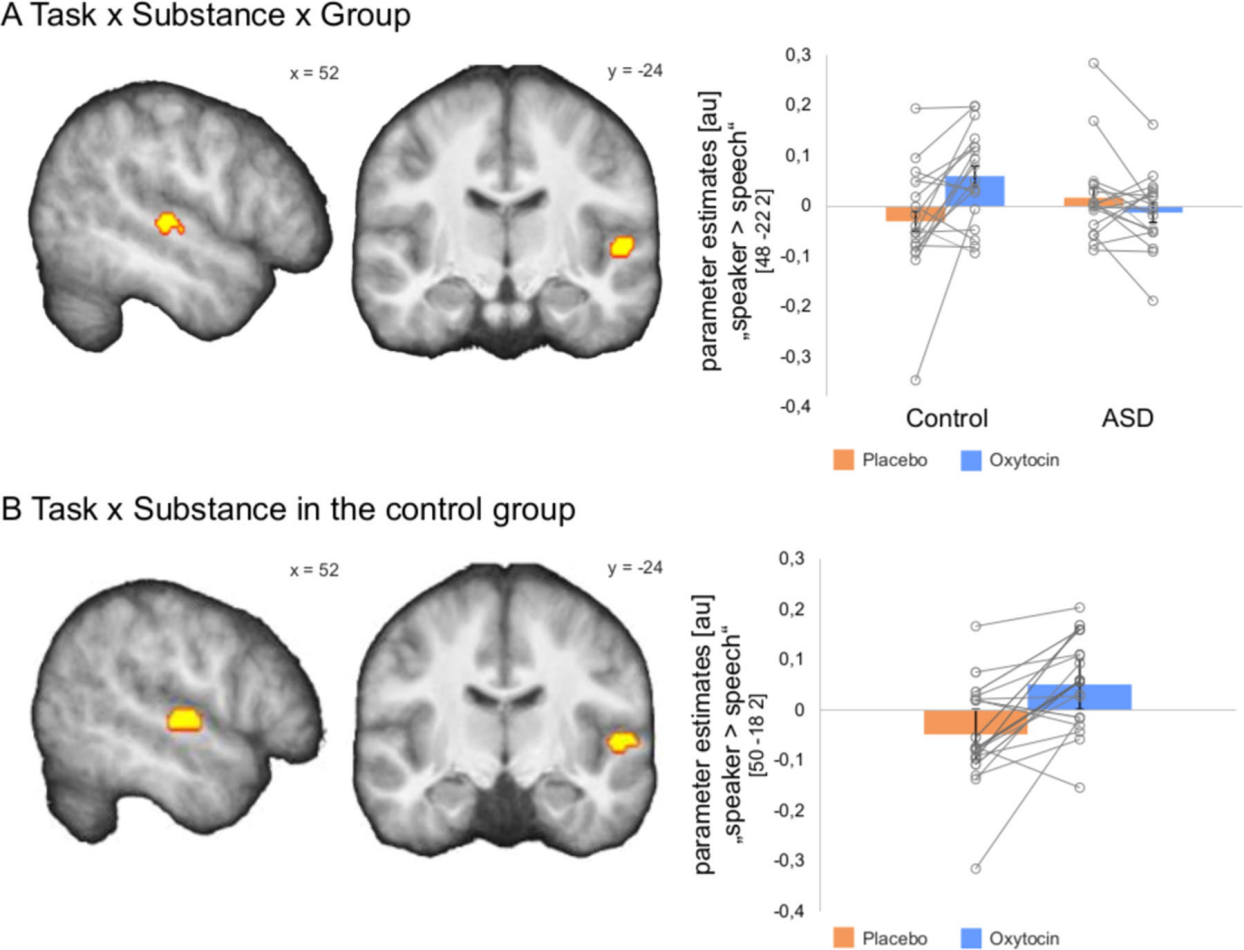
Effects of oxytocin compared to placebo on the right pSTS/G responses to the contrast “speaker > speech”. **a** A significant three-way interaction *Task* × *Substance* × *Group* revealed that BOLD responses in the right pSTS/G were modulated in the oxytocin compared to placebo condition differently in the control group than in the ASD group. Plots represent parameter estimates of the contrast “speaker > speech” extracted from the peak voxel of the significant interaction *Task* × *Substance* × *Group* for each substance condition and each group separately. **b** Post hoc analysis confirmed that oxytocin compared to placebo increased BOLD response in the right pSTS/G in the control group. Plots represent parameter estimates extracted from the peak voxel of the interaction *Task* × *Substance* in the control group for each substance condition separately. For display purposes only, effects are presented at the threshold of *p* = 0.01 uncorrected within the ROI. All results were overlaid onto a sample specific average image of normalized T1-weighted structural images. Plots display individual (i.e. circles) and mean-group (i.e. bars) results. Circles marking the same participant are connected with a line. x, y = MNI coordinates.

To test our main oxytocin-related hypotheses, we conducted a three-way ANOVA with within-subject factors *Task* (speaker, speech) and *Substance* (placebo, oxytocin), and the between-subject factor *Group* (control, ASD). We found a significant three-way interaction *Task* x *Substance* x *Group* (*p* = 0.006 FWE corrected; Fig. 3a; Supplementary Table S5), which indicated that oxytocin compared to placebo modulated the right pSTS/G responses to “speaker >speech” differently in the control group than in the ASD group. Extracting parameter estimates from the peak voxel of the significant interaction for each substance and group separately indicated that the interaction was driven by a response increase in the oxytocin compared to the placebo condition in the control group, but not in the ASD group (Fig. 3a; Supplementary Fig. S3A). There were no significant effects in other brain regions (*p* > 0.05 FWE corrected, whole brain).

Post hoc analysis revealed—as hypothesized—a significant interaction *Task* × *Substance* in the control group confirming that right pSTS/G responses increased in the oxytocin compared to placebo condition (*p* = 0.015 FWE corrected; Fig. 3b; Supplementary Fig. S3B; Supplementary Table S5; Supplementary Results—Control Analyses). There was, however, no significant *Task* × *Substance* interaction in the ASD group (*p* = 0.653 FWE corrected for the ROI).

### The ASD group had reduced right pSTS/G responses compared to controls

Next, we tested for replication of previous findings that in ASD compared to TD, responses to the contrast “speaker > speech” are reduced in the right pSTS/G^18^. The three-way ANOVA described in the previous section revealed a significant *Task* × *Group* interaction in the right pSTS/G (Fig. 2c; Supplementary Table S6). There were two clusters along the right pSTS/G: (1) within the second pSTS/G-ROI (*p* = 0.009 FWE corrected for the ROI) and (2) outside the pSTS/G-ROI (*p* = 0.004 FWE corrected, whole brain). Extracting parameter estimates from the peak voxels of the significant interaction for each group and substance separately indicated that this interaction was driven by higher right pSTS/G responses to “speaker > speech” in the control group compared to the ASD group across both substance conditions (Supplementary Fig. S4). In congruence with this interpretation, there was a main effect of *Task* in the control group, but not in the ASD group (see section “Right pSTS/G responses to speaker task compared to speech task in the control and ASD group”). These results are in agreement with our previous findings^18^. The *Task* × *Group* interaction was significant also in other brain regions for which we had no a priori hypotheses (*p* < 0.05 FWE corrected, whole brain; Supplementary Fig. S5; Supplementary Table S6).

The significant three-way interaction *Task* × *Substance* × *Group* described above (see section “Oxytocin increased right STS/G responses in controls, but not in ASD”) indicated that the group differences might be differently represented in the two substance conditions. Post hoc tests following the significant three-way interaction *Task* × *Substance* × *Group* revealed that the right pSTS/G responses were higher in the control than in the ASD group, but only in the oxytocin condition (*p* = 0.030 FWE corrected; Fig. 2c; Supplementary Table S6), and not in the placebo condition (*p* = 0.674 FWE corrected; Fig. 2c). Only at a lenient threshold, differences between the groups appeared in the placebo condition (*p* < 0.044 uncorrected).

### Correlation analysis

**Fig. 4:**
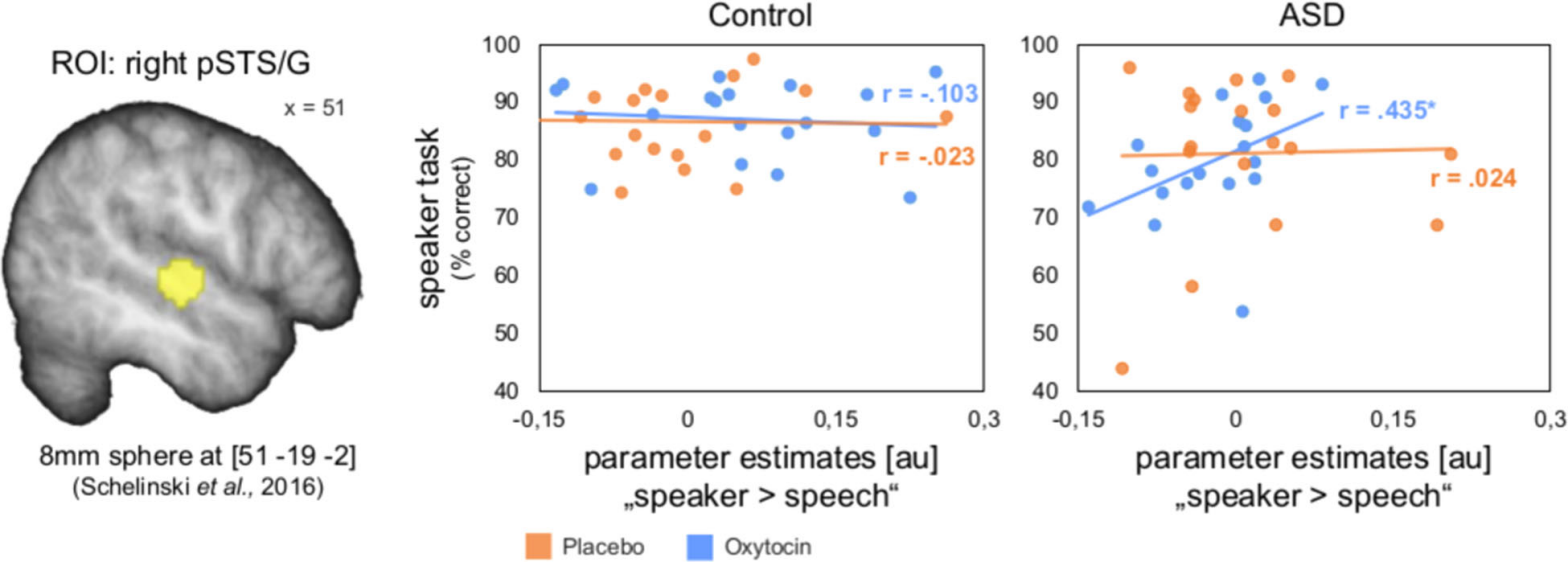
Correlation between right pSTS/G responses to the contrast “speaker > speech” and speaker recognition accuracy. In the ASD group, behavioral performance in the speaker task correlated significantly positively with parameter estimates of the contrast “speaker > speech” extracted from the right pSTS/G in the oxytocin condition (*p* = 0.036), while there was no such correlation in the placebo condition (*p* = 0.463). In the control, there was no such correlation in any of the substance conditions (both *p* values ≥ 0.342). The lines for each group represent the best-fitting linear regression. The pSTS/G sphere was overlaid onto a sample specific average image of normalized T1-weighted structural images. pSTS/G posterior superior temporal sulcus/gyrus. x = MNI coordinate.

In the ASD group, the right pSTS/G responses to “speaker > speech” were positively correlated with speaker recognition accuracy in the oxytocin condition (*r* = 0.435, *p* = 0.036, one-tailed) (Fig. 4). There was no such correlation in the placebo condition (*r* = 0.024, *p* = 0.463, one-tailed). In the control group, we found no significant positive correlation in the oxytocin condition (*r* = −0.103, *p* = 0.342, one-tailed) or in the placebo condition (*r* = −0.023, *p* = 0.463, one-tailed) (Supplementary Results—Correlation analysis).

There were no significant differences between the correlations in the oxytocin and in the placebo condition in the ASD group (z = 1.364, *p* = 0.086) or between the groups in the oxytocin condition (z = 1.559, *p* = 0.059), although both *p* values were close to the significance threshold. There were no significant differences between the correlations in the oxytocin and in the placebo condition in the control group (z = 0.314, *p* = 0.623), and no significant differences between the groups in the placebo condition (z = 0.129, *p* = 0.448).

## Discussion

The present study provided four key findings. First, intranasal administration of oxytocin compared to placebo increased the right pSTS/G responses to voice identity in contrast to speech recognition in TD individuals, but not in ASD. Second, in the ASD group, the right pSTS/G responses to voice identity compared to speech recognition were positively correlated with speaker recognition accuracy in the oxytocin, but not in the placebo condition. Third, behavioral speaker recognition accuracy was not altered by oxytocin in comparison to placebo in any of the groups. Fourth, we replicated previous findings about decreased right pSTS/G responses to voice-identity recognition in ASD compared to TD individuals. The findings corroborate the potential of intranasal oxytocin to enhance processing of communication signals in the human brain by demonstrating that oxytocin can boost brain responses to recognition of voice identity. However, this seemed to be the case only in TD individuals, but not in ASD, at least not on the group level. This result suggests that the oxytocin-related enhancement of voice-identity processing might be variable and dependent on functional integrity of the pSTS/G.

Several neuroimaging studies demonstrated that in TD individuals, intranasal oxytocin administration can modulate brain responses in visual sensory regions implicated in face perception^29–31^. Here, we showed that intranasal oxytocin can boost brain responses also in a voice-sensitive sensory brain region dedicated to perception of person identity from the voice^39,40^. To our knowledge, most previous studies on face and voice perception used emotional stimuli (e.g. refs. ^29,30,34^). In the present study, the increase of the right pSTS/G responses in TD controls was not driven by emotions as we used emotionally neutral voice stimuli. Moreover, previous studies compared social stimuli to non-social stimuli or to baseline conditions without stimulation^29,30,34^. In contrast, we found a response increase for a specific contrast between two conditions containing the same stimuli that required focusing on specific social information (i.e., speaker identity), while ignoring other social information (e.g., speech). Because there were no effects of intranasal oxytocin on speaker recognition accuracy in TD individuals, the oxytocin-related modulation of the right pSTS/G responses was not a result of an altered performance. Our findings, however, indicate that the mechanisms for voice-identity recognition can be changed by intranasal oxytocin. This suggests that TD individuals likely recruited different brain mechanisms under oxytocin in contrast to placebo to achieve a similar level of task performance.

A previous study demonstrated increased responses to emotional voices compared to a silent baseline condition in voice-sensitive sensory regions after oxytocin administration in adolescents with ASD^34^. In contrast, we found no significant oxytocin-related change in the right pSTS/G responses in the ASD group. This is in agreement with our prediction that atypical functioning of the right pSTS/G during voice-identity recognition might hinder the enhancement of its processing due to oxytocin. One such possible atypicality might be the organization of oxytocin receptors in the right pSTS/G to which intranasal oxytocin could bind. The availability and functioning of oxytocin receptors is coded by the oxytocin receptor gene (OXTR)^68^. In TD individuals, the OXTR expression in the STS/G is high^69–71^, indicating that this region is equipped with oxytocin receptors to which intranasal oxytocin can bind. In contrast, decreased OXTR gene expression was found in the temporal cortex (BA 41/42, BA 22) corresponding to the posterior portions of the STS/G in postmortem brains of individuals with ASD^72^. Therefore, the availability of OXTR in the pSTS/G in ASD might have modulated the responsiveness of this region to intranasal oxytocin. This could have contributed to more variable efficacy of intranasal oxytocin on the right pSTS/G responses in ASD and hence to an absence of a significant influence on the group level.

A lack of correspondence between oxytocin-related modulation of brain mechanisms and measurable behavioral changes on the group level, as in our controls, has been a common finding (e.g. refs. ^31,33^). This raises the question in how far enhanced brain responses to communication signal perception are reflected in improved behavioral performance. One possibility is that the present tasks were not sensitive enough to detect behavioral improvements. Previous studies reporting oxytocin-related enhancement of behavior investigated recognition of emotion from faces and voices^27,28^ or recognition of recently learned faces among novel faces^6,8^, while our design tested the ability to recognize recently learned voices among equally familiar voices. The other possibility is that oxytocin was not effective for improving recognition of voice identity, thus challenging the suitability of oxytocin to alleviate voice-identity recognition impairments in ASD in everyday life. Finally, the positive correlation between the right pSTS/G responses and the speaker recognition accuracy in the oxytocin condition in ASD suggested that the right pSTS/G responses were nevertheless modulated and systematically related to voice-identity recognition under oxytocin in ASD. On the one hand, it might be that oxytocin modulated voice-identity recognition positively, but only in some participants leading to an absence of its effect on the group level. On the other hand, the correlation could also be interpreted in a way that oxytocin reduced responses in the pSTS/G and concomitantly voice-identity recognition performance. Such interpretations are speculative at the moment, because correlation difference between the substance conditions in the ASD group was only close to significance.

The potential variability of oxytocin effects might be due to individual differences in the oxytocinergic system. One of such factors could be the functional integrity of the right pSTS/G region in terms of the availability and the functioning of oxytocin receptors to which intranasal oxytocin can bind. Variations of the OXTR have been also shown to influence the efficacy of intranasal oxytocin administration to enhance social behavior in TD individuals^73–75^ and to modulate brain responses to social tasks in TD individuals^76–78^ and in ASD^79^. These findings suggest that oxytocin efficacy may be genetically predicted before its actual administration and that only some participants will benefit from intranasal oxytocin. Moreover, single nucleoid polymorphisms in the OXTR have been associated with autistic symptoms, particularly within the social domain^80–82^ (but see ref. ^83^), suggesting potential differences in efficacy not only within groups, but also between the ASD and the TD population. It would be important to identify characteristics of potential treatment benefiters to better predict the efficacy of intranasal oxytocin in the future^84,85^.

The pSTS/G is a region characterized by a close proximity of auditory, visual, multisensory, social, and language processing (for review see refs. ^86–91^). Recently, the right pSTS/G was suggested to contain a modality-general representation of person identity (MNI coordinates of the centre of mass: *x* = 54, *y* = −44.5, *z* = 8)^91^. This specific region, however, does not overlap with the portions of the right pSTS/G where responses to voice-identity recognition were modulated by intranasal oxytocin in our study (22 mm posterior from the peak coordinate of the significant three-way interaction *Task* × *Substance* × *Group*). Moreover, our experiment was purely auditory as participants were familiarized with the speakers only by voice and not by face.

Sample size in our study was based on a previous study that found a significant difference between TD and ASD in the right pSTS/G responses in a similar voice-identity recognition fMRI experiment. Due to the heterogeneous nature of ASD, we specifically selected a subgroup of adults diagnosed with ASD without intellectual impairment to increase the homogeneity of ASD symptom characteristics. We accurately matched them pairwise to the TD participants to account for other sources of variability in behavior and brain responses. To reduce the likelihood of finding false positives we performed a hypothesis-driven ROI analysis^65,66^. We abstained from calculating effect sizes, as these can be overestimated in smaller sample sizes^92^. Future replication studies are required to corroborate current findings of the oxytocin-related modulation of voice-identity recognition in larger study samples^93^.

In conclusion, the present study showed that administration of intranasal oxytocin can enhance responses in the right pSTS/G during voice-identity recognition in TD individuals. In ASD, such an oxytocin-related modulation of voice-identity recognition is lacking across the whole group, potentially because of a variable level of functional integrity in the pSTS/G. The potential of intranasal oxytocin to enhance mechanisms for voice-identity recognition might be reliant on inter-individual characteristics of the pSTS/G.

## Supplementary information

Supplementary Information
