# Supplementary material for: Intranasal oxytocin modulates brain responses to voice-identity recognition in typically developing individuals, but not in ASD
Source: Transl Psychiatry. 2020 Jul 7;10:221. doi: 10.1038/s41398-020-00903-5 (PMC7341857; doi:10.1038/s41398-020-00903-5)
Supplement: Supplementary file 1 — Supplementary Information [file 41398_2020_903_MOESM1_ESM.doc]

**Supplementary Information**

**Supplementary Methods**

**Participants**

In total, we tested 20 participants with ASD (ASD group) and 20 pairwise matched controls (control group). Two participants with ASD were excluded from the study sample after data collection was completed: one due to difficulties in confirming the clinical diagnosis of ASD based on standard diagnostic criteria (ADOS, ADI-R; see below) and one due to a full-scale IQ score below normal range (lower than 85). Data of the respective control participants were excluded as well. Thus, the final study sample included 18 participants in each group.

IQ was assessed using the Wechsler Adult Intelligence Scale (WAIS). For 34 participants, we used WAIS-III ([1]; German adapted version: [2]). For one control and one ASD participant from different matching pairs, intelligence assessment was conducted within the context of another study using the WAIS-IV ([3]; German adapted version: [4]).

Moreover, one ASD participant scored on the Autism Spectrum Quotient below the cut-off value of 32 points (AQ score = 14) and lower than one of the control participants (AQ score = 23) [5]. This is predicted since the distribution of the AQ score overlaps between the ASD and the typically developing population [5]. The AQ is a self-assessment screening instrument for measuring the degree of autistic traits, but it does not serve as a diagnostic tool. It is suitable to discriminate between individuals diagnosed with ASD and typically developing controls (e.g., [5, 6]), but it does not significantly predict a receipt of ASD diagnosis [7].

**Experimental procedure**

The two fMRI sessions were separated by four weeks, because our study sample included six female participants (three ASD and three control participants). To rule out interactions of intranasal oxytocin with fluctuations of gonadal steroids over the menstrual cycle, all sessions were conducted during the luteal phase (self-reports), where endogenous oxytocin levels are lower compared to other phases of the menstrual cycle [8]. None of the female participants was taking hormonal contraceptives, was pregnant or breastfeeding during the study.

Participants received 24 International Units (IU) of oxytocin (Syntocinon-Spray, Novartis, Basel, Switzerland) or placebo via nasal spray. 45 minutes after substance administration, participants performed the voice-identity recognition fMRI experiment. Previous studies demonstrated that a similar dose and time schedule of intranasal oxytocin administration is suitable to modulate behavior and brain responses [9, 10]. The control and the ASD group received a comparable quantity of oxytocin and placebo spray measured as the weight of the administered spray (oxytocin condition: *t*(34) = .007, *p* = .994; placebo condition: (*t*(34) = .596, *p* = .555).

Nasal sprays and randomization of their administration were provided by the pharmacy of the University Hospital of Heidelberg. Study coordinator was unblinded to the randomization after data collection was completed.

***Mood questionnaire***

To assess potential nonspecific effects of substance administration on individual current state of the participants, we applied the MDBF after and before substance administration. It is a three-scale questionnaire assessing the current state on three scales (mood, wakefulness, calmness). Descriptive statistics can be found in Table S1.

For each scale separately, we conducted a repeated measures ANOVA with within-subject factors *Substance* (placebo, oxytocin) and *Time* (before substance administration, after substance administration), and the between-subject factor *Group* (control, ASD). For the mood scale and the wakefulness scale, we found no significant effects (all *p*s > .05). For the calmness scale, there was a main effect of *Group* (*F*(1,34) = 4.642, *p* = .038) and a significant interaction *Substance* x *Time* x *Group* (*F*(1,34) = 5.546, *p* = .024). Post hoc analysis using independent sample *t* tests revealed significant group differences in the calmness score before substance administration in the oxytocin condition (*t*(34) = 2.169, *p* = .037) and after substance administration in the placebo condition (*t*(34) = 2.679, *p* = .011). This indicates that the ASD group rated their calmness significantly lower than the control group. There were no further significant group differences.

**Voice-identity recognition experiment**

***Stimuli***

Stimulus material consisted of auditory-only two-word sentences spoken by eight professional male German native speakers (22, 22, 23, 25, 26, 28, 30, 31 years). The recorded sentences were semantically neutral, phonologically, and syntactically homogenous. Sentences consisted of the German pronoun “er” (English: “he”) and a German verb, e.g. “baut” (English: “builds”), each including a different verb. We instructed the speakers to read the sentences at a normal speech rate with an emotionally neutral intonation. All speakers were unfamiliar to the participants. The stimuli were recorded in a sound-attenuated chamber. For each speaker we used the same recording conditions and the same equipment (condenser microphone, Neumann TLM 50, Germany; preamplifier, Mic-Amp F-35; Lake People, Germany; soundcard, Power Mac G5 Dual 1.8 GHz; Apple Inc., USA; 44.1kHz sampling rate, and 16 bit resolution) and the software Sound Studio 3 (Felt Tip Inc., USA). Stimuli were post-processed (i.e., cutting, root mean square (rms) adjustment) using Audacity (version 1.3.5 beta; [http://audacity.sourceforge.net](http://audacity.sourceforge.net/)) and Matlab (version 7.7; The MathWorks, Inc., USA). Stimuli were presented using a MR confon system (Mark II; MR confon, Germany). Stimuli were presented and responses were recorded using Presentation software (version 16.3, Neurobehavioral Systems Inc., CA, USA).

Stimulus material was divided into two different stimulus sets and each stimulus set included 64 different sentences spoken by four speakers. In an independent pilot study, we ensured that both stimulus sets had a comparable difficulty level. For this purpose, we recruited 10 participants (3 male; *M*_Age_ = 22.40, *SD*_Age_ = 2.37), who were not included in the main study procedures involving oxytocin/ placebo administration. All participants were invited twice to perform two versions of the voice-identity recognition experiment with a two-week interval between the test sessions. Two additional participants were excluded from the sample, because they did not return to the lab for the second session. All participants were native German speakers. All reported normal hearing abilities and we confirmed these reports by means of pure tone audiometry (hearing level equal or below 25 dB at the frequencies 250 – 8000 Hz (Micromate 304; Madsen, Denmark). All participants were screened for presence of autistic traits and none of them met a clinically relevant extend as assessed by the AQ ([5]; *M*_AQ_ = 20.40, *SD*_AQ_ = 5.17)***.***

A repeated measures ANOVA with within-subject factors *Task* (speaker, speech) and *Test version* (version one, version two), and the between-subject factor *Presentation Order* (version one presented first, version two presented first) revealed no significant main effect of *Test Version* (*F*(1,8) = 1.354, *p* = .278), and no significant interaction *Task* x *Test version x Presentation order* (*F*(1,8) = .004, *p* = .954). This indicates that both versions of the voice-identity recognition fMRI experiment had a similar task difficulty.

***Experimental design***

Before performing the voice-identity recognition experiment in the MRI scanner, participants were familiarized with the speakers and the tasks on a laptop outside the MRI environment.

*Speaker familiarization*

In each session, all participants were familiarized with the four speakers’ voices that were subsequently presented in the voice-identity recognition fMRI experiment on a laptop outside the MRI-scanning room. The stimuli were presented via headphones (HD 201, Sennheiser, Germany). All participants listened to two-word sentences spoken in an emotionally neutral manner by the four speakers (e.g. “Er baut”, English: “He builds”). The sentences were organized in blocks of 10 sentences spoken by the same speaker. One sentence was presented for approximately 1 s. In total, there were two blocks per speaker and both blocks contained the same sentences. The order of speaker blocks was randomized across participants. Next, participants were tested on their ability to discriminate between the voices. For this purpose, pairs of sentences spoken by one or two of the four speakers were presented. Participants indicated whether the sentences were spoken by the same speaker or by two different speakers by pressing a corresponding button on the keyboard. Visual feedback about whether the given response was correct (green cross) or incorrect (red cross) was provided immediately after each response. In total, 128 trials were presented. In one trial (5 s), each sentence was presented for approximately 1 s and the response could be given until the end of the trial. Thus, the response window was 5 s long. Subsequently, visual feedback was presented for 2 s. The familiarization phase took approximately 20 min. The sentences used for the speaker familiarization were not repeated during the voice-identity experiment in the MRI scanner.

*Task familiarization*

Before the voice-identity recognition fMRI experiment, participants received task instructions and were familiarized with the speaker task and the speech task (two practice blocks per task) on a laptop outside the MRI scanner. In order to ensure that all participants understood the task equally well, the task familiarization was repeated if a participant performed less than 70% correct in the practice trials (repeated for 2 control participants and 12 ASD participants).

**MRI data acquisition and analysis**

Structural scans were recorded for co-registration purposes during image analysis. The structural scan was acquired using a 32-channel head coil and a T1-weighted 3D magnetization-prepared rapid gradient echo (MPRAGE) sequence (TR = 2300 ms, TE = 2.98 ms, TI=900 ms, flip angle = 9°, FOV = 256 mm x 240 mm, voxel size = 1 mm^3^ (isotropic resolution), 176 sagittal slices). This was done only for participants (n= 10) for whom no data was available from previous studies conducted at the Max Planck Institute for Human Cognitive and Brain Sciences in Leipzig. We accessed MPRAGE images available in the institute's data bank, which had been acquired also with a 32-channel coil and with the exact same acquisition parameters on 3Tesla MRI scanners (SIEMENS MAGNETOM Trio, Verio and Prisma (Siemens, Germany). To correct for field distortions, field-map scans were acquired consisting of a pair of 2D gradient echo images with different echo times (TE1/TE2 = 4.92ms/7.38ms) [11].

T2*- weighted images were spatially pre-processed using standard procedures: realignment and unwarp, normalization to Montreal Neurological Institute (MNI) standard stereotactic space using the T1 image of each participant, smoothing with an isotropic Gaussian filter of 8 mm at FWHM, and high-pass filtering at 128 s. Geometric distortions due to susceptibility gradients were corrected by an interpolation procedure based on the B0 field-map. To control for potential confounding effects of movement artefacts on the BOLD signal change we examined the head movement along six possible axes (translation along x-axis, translation along y-axis, translation along z-axis, rotation around x-axis, rotation around y-axis and rotation around z-axis) during the voice-identity recognition fMRI experiment. We extracted six movement parameters resulting from rigid body transformation during spatial realignment (Table S2). We conducted a three-way ANOVA with the within-subject factors *Substance* (placebo, oxytocin) and *Axis* (translation x-axis, translation y-axis, translation z-axis, rotation x-axis, rotation y-axis, rotation z-axis), and the between-subject factor *Group* (control, ASD). We found no significant main effect of *Group* (*p =* .385) and no significant interaction *Substance* x *Group* (*p* = .778) showing that head movement was not significantly different between the groups and the substance conditions (Table S2).

At the first level, statistical parametric maps were generated by modeling the evoked hemodynamic response for the different conditions as boxcars convolved with a synthetic hemodynamic response function within general linear model [12]. We modeled the conditions “speaker”, “speech” and “instruction”. Head movement parameters were modeled as covariates of no interest.

At the second level, population-level inferences about BOLD signal changes were based on a random effects model that estimated the second-level statistic at each voxel. To assess the differences between the groups and between the substances, we employed a flexible factorial design as implemented in SPM12. We performed a three-way ANOVA with the within-subject factors *Task* (speaker, speech) and *Substance* (placebo, oxytocin), and the between-subject factor *Group* (control, ASD) including single-participant contrast images from both groups.

**Control analyses**

We conducted two control analyses. In the first control analysis, we aimed to ensure that the potential lack of significant modulation of the right pSTS/G responses to “speaker > speech” in the ASD group was not due to the choice of the right pSTS/G-ROI. For this, we applied the first pSTS/G-ROI (Figure S1A) that included more extensive portions of the right pSTS/G in comparison to the second right pSTS/G-ROI that was used in the main second-level analysis (Figure S1B). In the second control analysis, we examined if administration of oxytocin compared to placebo modulated the right pSTS/G responses to the contrasts “speaker > silent baseline” and “speech > silent baseline”. We did this because Gordon et al. [13] reported an increase of responses to emotional voices compared to silent baseline condition in auditory sensory regions including the bilateral STS/G in ASD.

**Supplementary Results**

**Response times**

A repeated measures ANOVA on the response times with within-subject factors *Task* (speaker, speech) and *Substance* (placebo, oxytocin), and the between-subject factor *Group* (control, ASD) revealed a main effect of Task indicating that both groups showed significantly higher response times in the speech task compared to the speaker task (*F*(1,36) = 176.152, *p* < .001) (Figure S2B, Table S3). No other effects reached significance (all *p*s > .05).

**Correlation analysis**

The significant positive correlation in the ASD group between right pSTS/G responses to “speaker > speech” and behavioral speaker recognition accuracy was not driven by potential outliers and robust to their removal. We ensured this by excluding two data points which were either two standard deviations away from the mean parameter estimate of the “speaker > speech” contrast or from the mean recognition accuracy in the speaker task. The re-calculated correlation remained significant (*r* = .649, *p* = .003). Therefore, the analysis was continued including all data points.

**Control analyses**

***Control analysis 1: Right pSTS/G responses to “speaker > speech”***

Results of the control analysis using the first pSTS/G-ROI were almost identical to the results of the main analysis conducted with the second pSTS/G-ROI. This confirmed that the increase of the right pSTS/G responses in the control group and the lack of it in the ASD group were independent of the ROI definition. The three-way interaction *Task* x *Substance* x *Group* was significant indicating that oxytocin compared to placebo differentially influenced the right pSTS/G responses in the control group and in the ASD group (x = 48, y = -22, z = 2; *p* = .018 FWE corrected for the ROI). Post hoc analyses in each group separately revealed a significant interaction *Task* x *Substance* in the control group (x = 50, y = -18, z = 2; *p* = .041 FWE corrected), but not in the ASD group (*p* = .048 uncorrected).

***Control analysis 2: Right pSTS/G responses to contrasts “speaker > silent baseline” and “speech > silent baseline”***

We assessed if administration of oxytocin compared to placebo modulated the right pSTS/G responses to the contrasts “speaker > silent baseline” and “speech > silent baseline”. For each contrast separately, we conducted a repeated measures ANOVA with within-subject factors *Task* (speaker/ speech, baseline) and *Substance* (placebo, oxytocin), and the between-subject factor *Group* (control, ASD).

*Oxytocin did not modulate right pSTS/G responses to “speaker > silent baseline”:* The three-way ANOVA revealed no main effect of *Substance* (both *p* values ≥ .247 FWE corrected) and no three-way interaction of *Task* x *Substance* x *Group* (both *p* values ≥. 344 FWE corrected) indicating that oxytocin compared to placebo did not influence the right pSTS/G responses to the speaker vs. silent baseline in any of the groups.

*Oxytocin did not modulate the right pSTS/G responses to ”speech task > silent baseline”:* The three-way ANOVA revealed no main effect of *Substance* (both *p* values ≥ .142 FWE corrected) and no three-way interaction of *Task* x *Substance* x *Group* (both *p* values ≥ .131 FWE corrected) indicating that oxytocin compared to placebo did not influence the right pSTS/G responses to speech vs. silent baseline in any of the groups.

**Supplementary References**

1. Wechsler D. *Wechsler Adult Intelligence Scale (WAIS-III).* The Psychological Corporation: San Antonio, USA, 1997
2. von Aster M, Neubauer A, Horn R. *Wechsler Intelligenztest Für Erwachsene (WIE).* Harcourt Test Services: Frankfurt/Main, Germany, 2006.
3. Wechsler D. *Wechsler Adult Intelligence Scale–Fourth Edition (WAIS–IV).* NCS Pearson: San Antonio, USA, 2008.
4. Petermann F. *Wechsler Adult Intelligence Scale (WAIS‐IV, German Version).* Pearson Assessment and Information: Frankfurt/Main, Germany, 2012.
5. Baron-Cohen S, Wheelwright S, Skinner R, Martin J, Clubley E. The autism-spectrum quotient (AQ). Evidence from Asperger syndrome/high-functioning autism, males and females, scientists and mathematicians. J Autism Dev Disord 2001; **31**: 5-17.
6. Wakabayashi A, Baron-Cohen S, Wheelwright S, Tojo Y. The Autism-Spectrum Quotient (AQ) in Japan: a cross-cultural comparison. J Autism Dev Disord 2006; **36**: 263-270.
7. Ashwood KL et al. Predicting the diagnosis of autism in adults using the Autism-Spectrum Quotient (AQ) questionnaire. Psychol Med 2016; **46**: 2595-2604.
8. Salonia A et al. Menstrual cycle-related changes in plasma oxytocin are relevant to normal sexual function in healthy women. Horm Behav 2005; **47**: 164-169.
9. Heinrichs M, von Dawans B, Domes G. Oxytocin, vasopressin, and human social behavior. Front Neuroendocrinol 2009; **30**: 548-557.
10. MacDonald K, MacDonald TM. The peptide that binds: a systematic review of oxytocin and its prosocial effects in humans. Harv Rev Psychiatry 2010; **18**: 1-21.
11. Jezzard P, Balaban RS. Correction for geometric distortion in echo planar images from B0 field variations. Magn Reson Med 1995; **34**: 65-73.
12. Friston KJ, Ashburner A, Kiebel S, Nichols T, Penny W (Eds). *Statistical Parametric Mapping, The Analysis of Functional Brain Images.* Academic Press, 2007
13. Gordon I et al. Intranasal oxytocin enhances connectivity in the neural circuitry supporting social motivation and social perception in children with autism. Sci Rep 2016; **6**: 35054.
14. Blank H, Anwander A, von Kriegstein K. Direct structural connections between voice-and face-recognition areas. J Neurosci 2011; **31**: 12906-12915.
15. Schelinski S, Borowiak K, von Kriegstein K. Temporal voice areas exist in autism spectrum disorder but are dysfunctional for voice identity recognition. Soc Cogn Affect Neurosci 2016; **11**: 1812-1822.
16. Roswandowitz C, Schelinski S, von Kriegstein K. Developmental phonagnosia: linking neural mechanisms with the behavioural phenotype. NeuroImage 2017; **155**: 97-112.
17. Desikan RS et al*.* An automated labeling system for subdividing the human cerebral cortex on MRI scans into gyral based regions of interest. Neuroimage 2006; **31**: 968-980.
18. Smith SM et al. Advances in functional and structural MR image analysis and implementation as FSL. Neuroimage. 2004; **23**: S208-S219.
19. Eickhoff SB et al. Assignment of functional activations to probabilistic cytoarchitectonic areas revisited. Neuroimage 2007; **36**: 511-521.

**Supplementary Figures**


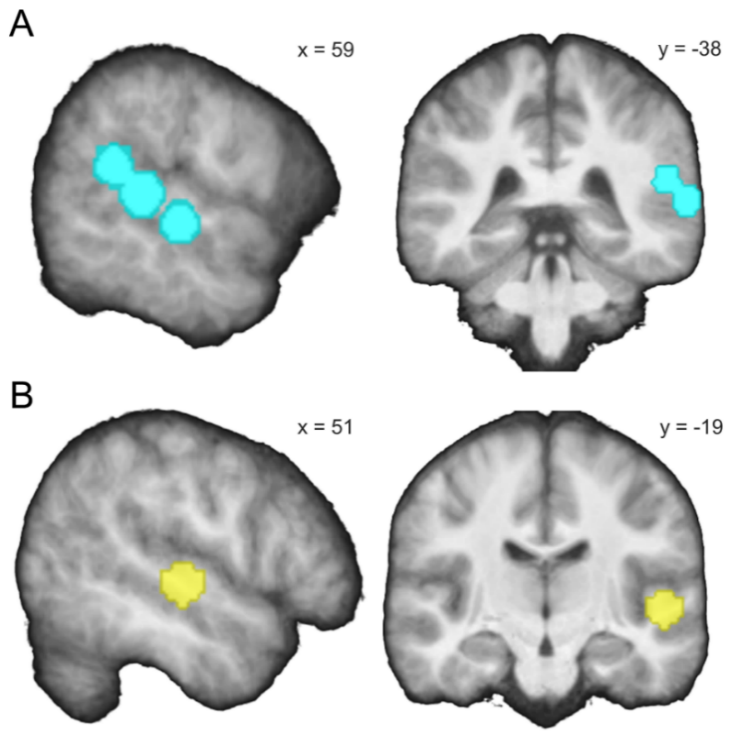


**Figure S1.** Regions of interest in the right pSTS/G. **(A)** The first right pSTS/G ROI included portions of the right pSTS/G that had been previously shown to respond to the contrast “speaker > speech” in TD individuals [14-16]. **(B)** The second right pSTS/G ROI included portions of the right pSTS/G that had been previously reported to have reduced responses to the contrast “speaker > speech” in ASD compared to typically developing individuals [15]. All ROIs are overlaid onto a sample specific average image of normalized T1-weighted structural images of all participants in the study (n = 36). x, y = MNI coordinates.

**
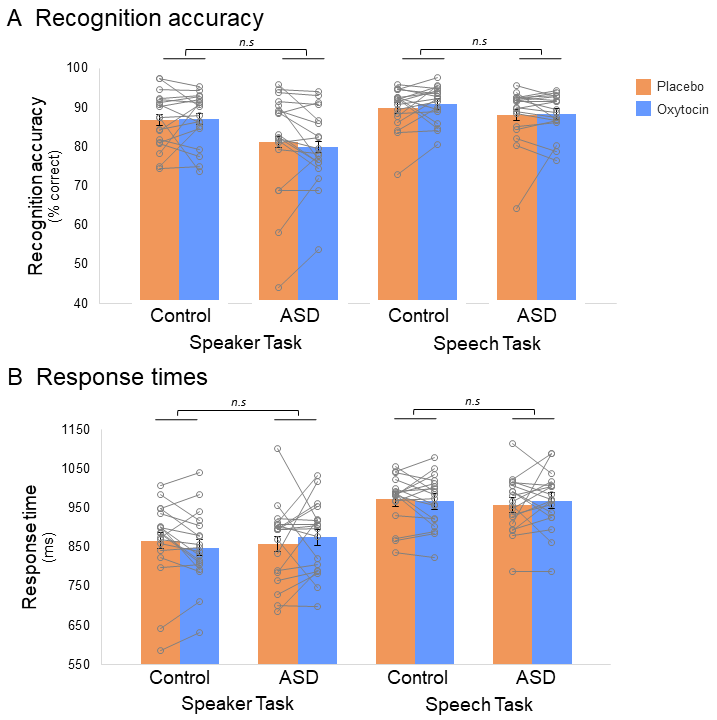
**

**Figure S2.** Behavioral performance in the voice-identity recognition experiment. **(A)** Recognition accuracy measured in % correct recognition. There were no significant effects of oxytocin compared to placebo administration in either of the groups. Only in the oxytocin condition, the ASD group performed significantly worse in speaker task than the control group. There were no such group differences in the speaker task in the placebo condition, or in the speech task in any of the substance conditions. **(B)** Response times measured in milliseconds (ms). Both groups responded significantly faster in the speaker task compared to the speech task, independent of the substance condition. There were no significant differences between oxytocin and placebo administration or between the ASD and the control group. We display individual (i.e. circles) and mean-group (i.e. bars) results. Circles marking the same participant are connected with a line. Error bars represent +/- 1 SE; ** *p* < .001; * *p* < .05; *ns*. = not significant.

**
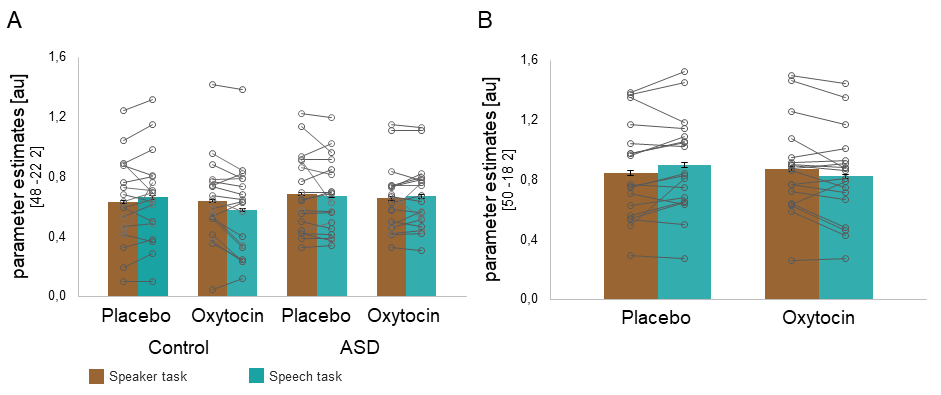
**

**Figure S3** Effects of oxytocin compared to placebo on the right pSTS/STG responses to the contrast “speaker > speech”. **(A)** Plots represent parameter estimates extracted from the peak voxel of the interaction *Task* x *Substance* x *Group* for each substance, task condition and group separately. **(B)** Plots represent parameter estimates extracted from the peak voxel of the interaction *Task* x *Substance* in the control group for each substance and task condition separately. We display individual (i.e. circles) and mean-group (i.e. bars) results. Circles marking the same participant are connected with a line. Error bars represent +/- 1 SE.


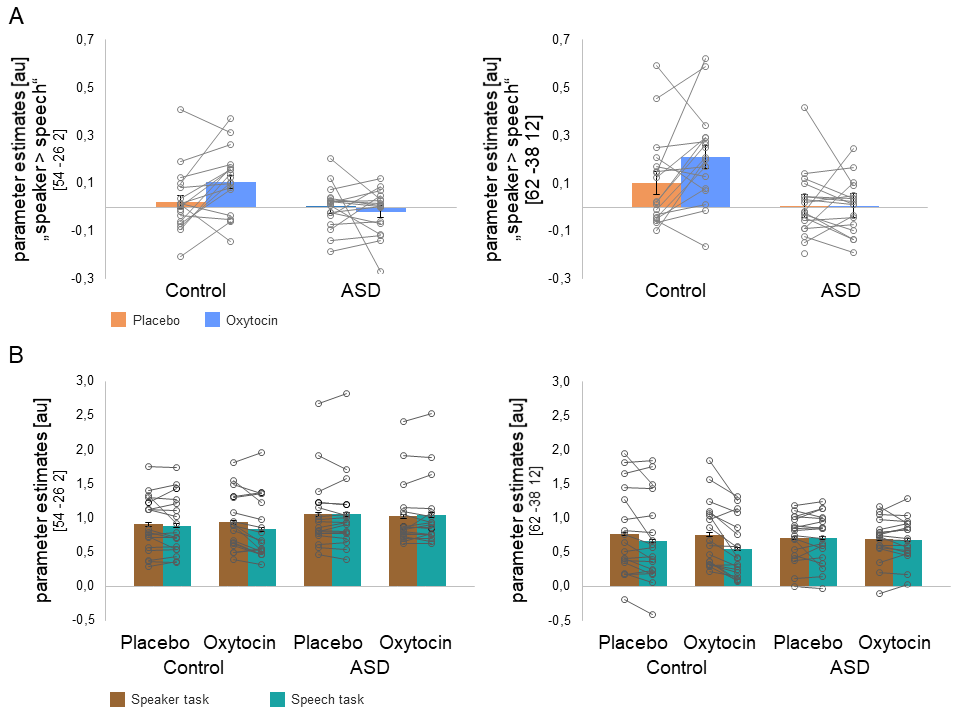


**Figure S4.** Group differences in the right pSTS/STG responses to the contrast “speaker > speech”. **(A)** Plots represent parameter estimates extracted from the peak voxels of the interaction *Task* x *Group* for each substance condition and group separately. **(B)** Plots represent parameter estimates extracted from peak voxels of the interaction *Task* x *Group* for each substance, task condition and group separately. We display individual (i.e. circles) and mean-group (i.e. bars) results. Circles marking the same participant are connected with a line. Error bars represent +/- 1 SE.

**
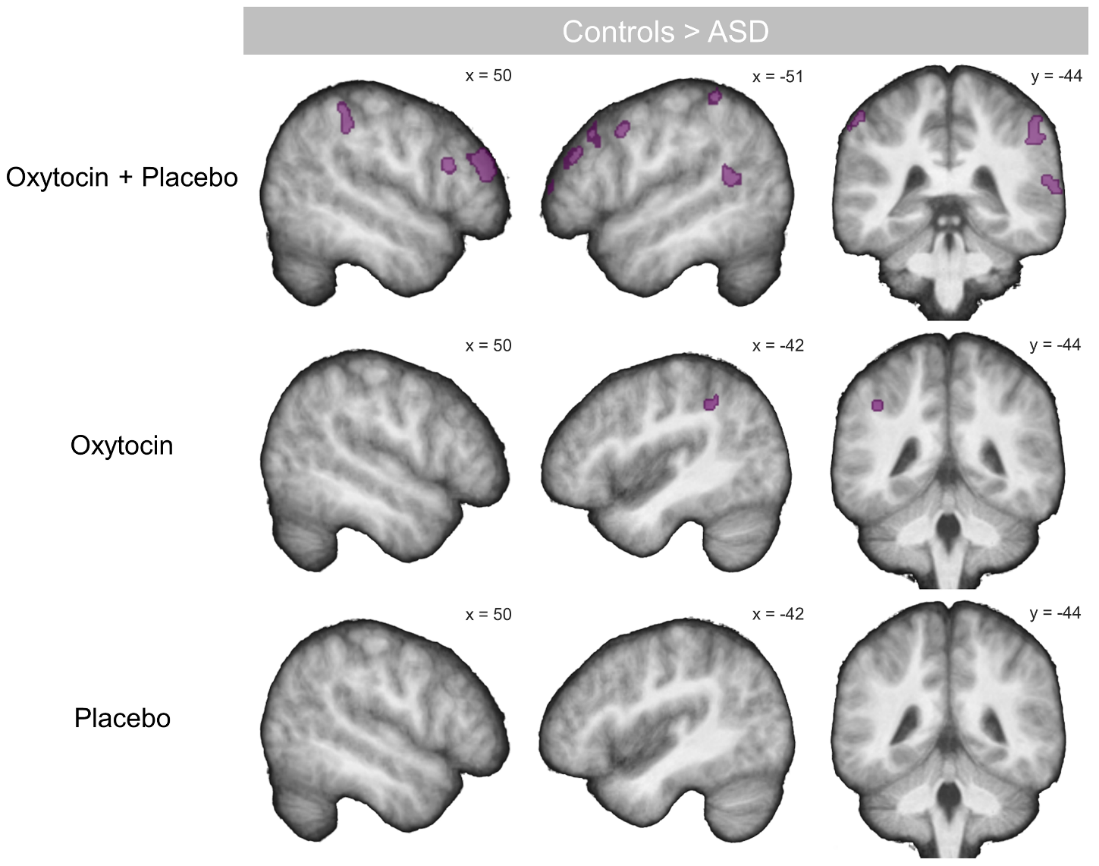
**

**Figure S5.** Group differences in responses to the contrast “speaker > speech” in brain regions other than the right pSTS/G. **(A)** Across both substance conditions, the control group compared to the ASD group showed significantly higher BOLD responses when recognizing speaker compared to speech. **(B)** In the oxytocin condition, the control group compared to the ASD group showed significantly higher BOLD responses when recognizing speaker compared to speech in the left supramarginal gyrus. **(C)** In the placebo condition, there were no significant group differences. The effects are significant at *p* < .05 FWE corrected at the whole-brain level. For display purposes only, within-group effects are presented at the threshold of *p* = .01 uncorrected within the ROI and between-group effects at the threshold of *p* = .05 uncorrected within the ROI. All results were overlaid onto a sample specific average image of normalized T1-weighted structural images. x, y = MNI coordinates.

**Supplementary Tables**

**Table S1.** Summary of average scores in the mood questionnaire (Steyer et al., 1997).

|  |  | **Control** | | | | **ASD** | | | |
| --- | --- | --- | --- | --- | --- | --- | --- | --- | --- |
|  |  | ***Placebo*** | | ***Oxytocin*** | | ***Placebo*** | | ***Oxytocin*** | |
|  | ***Time*** | ***M*** | ***SD*** | ***M*** | ***SD*** | ***M*** | ***SD*** | ***M*** | ***SD*** |
| Good-Bad Mood | 1 | 16.50 | 2.33 | 16.83 | 2.15 | 16.44 | 2.43 | 15.61 | 2.64 |
|  | 2 | 16.72 | 2.22 | 16.78 | 1.98 | 15.56 | 2.33 | 16.06 | 2.07 |
| Alertness-Tiredness | 1 | 14.89 | 2.85 | 15.00 | 2.74 | 14.00 | 3.79 | 14.67 | 4.13 |
|  | 2 | 14.06 | 3.56 | 13.00 | 3.34 | 14.00 | 3.65 | 14.72 | 3.48 |
| Calmness-  Restlessness | 1 | 16.39 | 2.62 | 16.72 | 2.11 | 14.89 | 2.81 | 14.61 | 3.55 |
|  | 2 | 17.28 | 2.24 | 16.44 | 2.75 | 14.56 | 3.68 | 15.39 | 3.09 |

*M* = mean; *SD* = standard deviation.

**Table S2.** Group comparison of the maximum head movement (in mm) for all possible directions (x, y and z).

|  | **Control** | | | | **ASD** | | | |
| --- | --- | --- | --- | --- | --- | --- | --- | --- |
|  | ***Placebo*** | | ***Oxytocin*** | | ***Placebo*** | | ***Oxytocin*** | |
|  | ***M*** | ***SD*** | ***M*** | ***SD*** | ***M*** | ***SD*** | ***M*** | ***SD*** |
| Translation X | .368 | .172 | .434 | .249 | .496 | .360 | .658 | .916 |
| Translation Y | .392 | .194 | .677 | .688 | .606 | .542 | .930 | 1.388 |
| Translation Z | 1.016 | .493 | 1.363 | .944 | 1.228 | .816 | 1.187 | 1.115 |
| Rotation X | .013 | .008 | .018 | .019 | .017 | .0157 | .019 | .016 |
| Rotation Y | .008 | .004 | .010 | .004 | .011 | .006 | .013 | .011 |
| Rotation Z | .007 | .004 | .007 | .005 | .008 | .008 | .011 | .021 |

*M* = mean; *SD* = standard deviation.

**Table S3.** Summary of behavioral performance in the voice-identity recognition fMRI experiment.

| **Recognition accuracy** | | | | | | | | |
| --- | --- | --- | --- | --- | --- | --- | --- | --- |
|  | **Control** | | | | **ASD** | | | |
|  | ***Placebo*** | | ***Oxytocin*** | | ***Placebo*** | | ***Oxytocin*** | |
|  | ***M*** | ***SD*** | ***M*** | ***SD*** | ***M*** | ***SD*** | ***M*** | ***SD*** |
| Speaker task | 86.70 | 7.11 | 87.04 | 6.73 | 81.19 | 13.62 | 79.98 | 9.90 |
| Speech task | 89.77 | 5.54 | 90.97 | 4.57 | 88.17 | 7.26 | 88.26 | 5.27 |
| **Response times** | | | | | | | | |
|  | ***M*** | ***SD*** | ***M*** | ***SD*** | ***M*** | ***SD*** | ***M*** | ***SD*** |
| Speaker task | 865.89 | 106.34 | 848.58 | 94.19 | 858.05 | 101.92 | 874.31 | 92.50 |
| Speech task | 972.09 | 60.82 | 966.77 | 66.02 | 957.40 | 71.76 | 968.34 | 74.39 |

M = mean; SD = standard deviation.

**Table S4.** Coordinates for significant BOLD responses in the right pSTS/G to “speaker > speech”.

| **Placebo + Oxytocin** | | | | | | | |
| --- | --- | --- | --- | --- | --- | --- | --- |
| ***Control*** | | | | ***ASD*** | | | |
| ***x*** | ***y*** | ***z*** | ***Z*** | ***x*** | ***y*** | ***z*** | ***Z*** |
| ^a^58 | -40 | 18 | 4.81 | - | | | |
| 52 | -50 | 18 | 3.42 |  |  |  |  |
| **Placebo** | | | | | | | |
| ***x*** | ***y*** | ***z*** | ***Z*** | ***x*** | ***y*** | ***z*** | ***Z*** |
| ^a^58 | -40 | 18 | 3.13 | - | | | |
| **Oxytocin** | | | | | | | |
| ***x*** | ***y*** | ***z*** | ***Z*** | ***x*** | ***y*** | ***z*** | ***Z*** |
| ^a^62 | -38 | 12 | 3.89 | - | | | |

Coordinates represent local response maxima in MNI space (in mm). Coordinates are reported that reached significance at *p* < .05 FWE corrected (peak-level) within the respective ROI and which cluster size contained more than 5 voxels. Regions were labelled using a standard anatomical atlas (Harvard-Oxford cortical and subcortical structural atlases; [17]) implemented in FSL ([18]; http://www.fmrib.ox.ac.uk/fsl/fslview). pSTS/G = posterior Superior Temporal Sulcus/ Gyrus; *Z* indicates the statistical value. ^a^ peak in the Supramarginal Gyrus.

**Table S5.** Effects of substance application on the right pSTS/G responses to “speaker > speech”.

| **Substance differences** | | | | | | | |
| --- | --- | --- | --- | --- | --- | --- | --- |
| ***(Oxytocin > Placebo) x (Control + ASD)*** | | | | ***(Placebo > Oxytocin) x (Control + ASD)*** | | | |
| ***x*** | ***y*** | ***z*** | ***Z*** | ***x*** | ***y*** | ***z*** | ***Z*** |
| - | | | | - | | | |
| ***(Oxytocin > Placebo) x (Control > ASD)*** | | | | ***(Oxytocin > Placebo) x (ASD > Control)*** | | | |
| 48 | -22 | 2 | 3.49 | - | | | |
| ***(Oxytocin > Placebo) x Control*** | | | | ***(Oxytocin > Placebo) x ASD*** | | | |
| ^a^50 | -18 | 2 | 3.26 | *-* | | | |
| 58 | -18 | 2 | 2.86 |  |  |  |  |

Coordinates represent local response maxima in MNI space (in mm). Coordinates are reported that reached significance at *p* < .05 FWE corrected (peak-level) within the respective ROI and which cluster size contained more than 5 voxels. Regions were labelled using a standard anatomical atlas (Harvard-Oxford cortical and subcortical structural atlases; [17]) implemented in FSL ([18]; http://www.fmrib.ox.ac.uk/fsl/fslview). pSTS/G = posterior Superior Temporal Sulcus/ Gyrus; *Z* indicates the statistical value. *Z* indicates the statistical value. ^a^ peak in the Heschl’s Gyrus.

**Table S6.** Group differences in BOLD responses to “speaker > speech”.

|  |  | ***Placebo + Oxytocin*** | | | | | | | | | | | |
| --- | --- | --- | --- | --- | --- | --- | --- | --- | --- | --- | --- | --- | --- |
|  |  | ***Control > ASD*** | | | | | | | ***ASD > Control*** | | | | |
| ***Region*** |  | ***x*** | ***y*** | | ***z*** | | ***Z*** | | ***x*** | | ***y*** | ***z*** | ***Z*** |
| pSTS/G ROI |  | 54 | | -26 | | -1 | | 3.38 | | - | | | |
| pSTS/G |  | ^a^62 | | -38 | | 12 | | 5.21 | | - | | | |
|  |  | 62 | | -42 | | 12 | | 4.64 | |  | | | |
| IFG^a^ | l | -56 | | 8 | | 37 | | 6.15 | | - | | | |
|  |  | -54 | 20 | | 32 | | 5.41 | |  | |  |  |  |
|  |  | -58 | 18 | | 21 | | 5.18 | |  | |  |  |  |
|  | l | -52 | 32 | | 21 | | 5.09 | |  | |  |  |  |
| SPL | l | -28 | -56 | | 40 | | 5.88 | |  | |  |  |  |
|  |  | -28 | -66 | | 48 | | 4.84 | |  | |  |  |  |
|  |  | -40 | -50 | | 43 | | 4.79 | |  | |  |  |  |
|  | r | 42 | -50 | | 59 | | 5.29 | |  | |  |  |  |
|  | r | 38 | -48 | | 43 | | 5.11 | |  | |  |  |  |
| SMG | l | -54 | -40 | | 54 | | 5.79 | |  | |  |  |  |
|  | r | 52 | -36 | | 40 | | 5.32 | |  | |  |  |  |
| MTG | l | -48 | -50 | | 10 | | 5.59 | |  | |  |  |  |
| Frontal Pole | r | 50 | 36 | | 15 | | 5.52 | |  | |  |  |  |
|  |  | 48 | 40 | | 26 | | 5.30 | |  | |  |  |  |
|  |  | 56 | 16 | | 15 | | 5.29 | |  | |  |  |  |
|  | l | -50 | 44 | | 7 | | 4.99 | |  | |  |  |  |
| PaCG | r | 2 | 16 | | 48 | | 5.19 | |  | |  |  |  |
| LOC | l | -32 | -72 | | 37 | | 5.09 | |  | |  |  |  |
| Occipital Pole | r | 20 | -92 | | 2 | | 4.87 | |  | |  |  |  |
| Cerebellum | l | -8 | -78 | | -29 | | 4.82 | |  | |  |  |  |
|  |  | ***Placebo*** | | | | | | | | | | | |
| ***Region*** |  | ***x*** | ***y*** | | ***z*** | | ***Z*** | | ***x*** | | ***y*** | ***z*** | ***Z*** |
|  |  | - | | | | | | | - | | | | |
|  |  | ***Oxytocin*** | | | | | | | | | | | |
| ***Region*** |  | ***x*** | ***y*** | | ***z*** | | ***Z*** | | ***x*** | | ***y*** | ***z*** | ***Z*** |
| pSTS/G ROI |  | ^a^58 | -18 | | 2 | | 2.96 | | - | | | | |
| SMG | l | -42 | -44 | | 37 | | 5.11 | | - | | | | |

Coordinates represent local response maxima in MNI space (in mm). Coordinates in the right pSTS/G ROI are reported that reached significance at *p* < .05 FWE corrected for the ROI (peak-level). Otherwise, coordinates are reported that reached significance at *p* < .05 FWE corrected (peak-level) at the whole-brain level, and which cluster size contained more than 5 voxels. Regions were labelled using a standard anatomical atlas (Harvard-Oxford cortical and subcortical structural atlases; [17]) and Jülich histological (cyto- and myeloarchitectonic) atlas; [19]), implemented in FSL ([18]; http://www.fmrib.ox.ac.uk/fsl/fslview). pSTS/G = posterior Superior Temporal Sulcus/ Gyrus; ROI = Region of Interest; IFG = Inferior Frontal Gyrus; SPL = Superior Parietal Lobule; SMG = Supramarginal Gyrus; MTG = Middle Temporal Gyrus; PaCG = Paracingulate Gyrus; LOC = Lateral Occipital Cortex. *Z* indicates the statistical value. ^a^ peak in the Precentral Gyrus; ^b^ peak in the Frontal Pole.
